# The Three Paralogous MicroRNA Clusters in Development and Disease, miR-17-92, miR-106a-363, and miR-106b-25

**DOI:** 10.1155/2016/1379643

**Published:** 2016-04-04

**Authors:** Cuong Khuu, Tor Paaske Utheim, Amer Sehic

**Affiliations:** ^1^Department of Oral Biology, Faculty of Dentistry, University of Oslo, 0372 Oslo, Norway; ^2^Department of Medical Biochemistry, Oslo University Hospital, 0407 Oslo, Norway; ^3^Department of Ophthalmology, Drammen Hospital, Vestre Viken Hospital Trust, 3004 Drammen, Norway; ^4^Faculty of Health Sciences, University College of South East Norway, 3614 Kongsberg, Norway

## Abstract

MicroRNAs (miRNAs) form a class of noncoding RNA genes whose products are small single-stranded RNAs that are involved in the regulation of translation and degradation of mRNAs. There is a fine balance between deregulation of normal developmental programs and tumor genesis. An increasing body of evidence suggests that altered expression of miRNAs is entailed in the pathogenesis of human cancers. Studies in mouse and human cells have identified the miR-17-92 cluster as a potential oncogene. The miR-17-92 cluster is often amplified or overexpressed in human cancers and has recently emerged as the prototypical oncogenic polycistron miRNA. The functional analysis of miR-17-92 is intricate by the existence of two paralogues: miR-106a-363 and miR-106b-25. During early evolution of vertebrates, it is likely that the three clusters commenced via a series of duplication and deletion occurrences. As miR-106a-363 and miR-106b-25 contain miRNAs that are very similar, and in some cases identical, to those encoded by miR-17-92, it is feasible that they regulate a similar set of genes and have overlapping functions. Further understanding of these three clusters and their functions will increase our knowledge about cancer progression. The present review discusses the characteristics and functions of these three miRNA clusters.

## 1. Introduction

Embryonic development in vertebrates is carefully orchestrated and requires tightly regulated gene expression processes. It is increasingly evident that miRNAs constitute an essential role in vertebrate development, as documented by the early embryonic lethality of mice with defects in the miRNA biogenesis pathway [1–3]. Over the past decade, miRNAs have emerged as important players in RNA interference-mediated posttranscriptional gene regulation. MicroRNAs are a class of non-protein-coding RNAs (~22 nt in length), which are essential to normal cellular physiology, including development and proliferation [4]. MicroRNAs provide a convenient and efficient pathway for regulation of gene expression at a posttranscriptional level. It is believed that miRNAs regulate the expression of about 30% of protein-coding genes by targeting their mRNAs, causing either mRNA cleavage or inhibition of translation [5–7]. This is brought about by the partially complementary pairing of miRNA to the 3′ untranslated regions (UTRs) of its target mRNA using the seed region (positions 2 to 7 or 8 from 5′-end) of the miRNAs [8].

**Figure 1:**
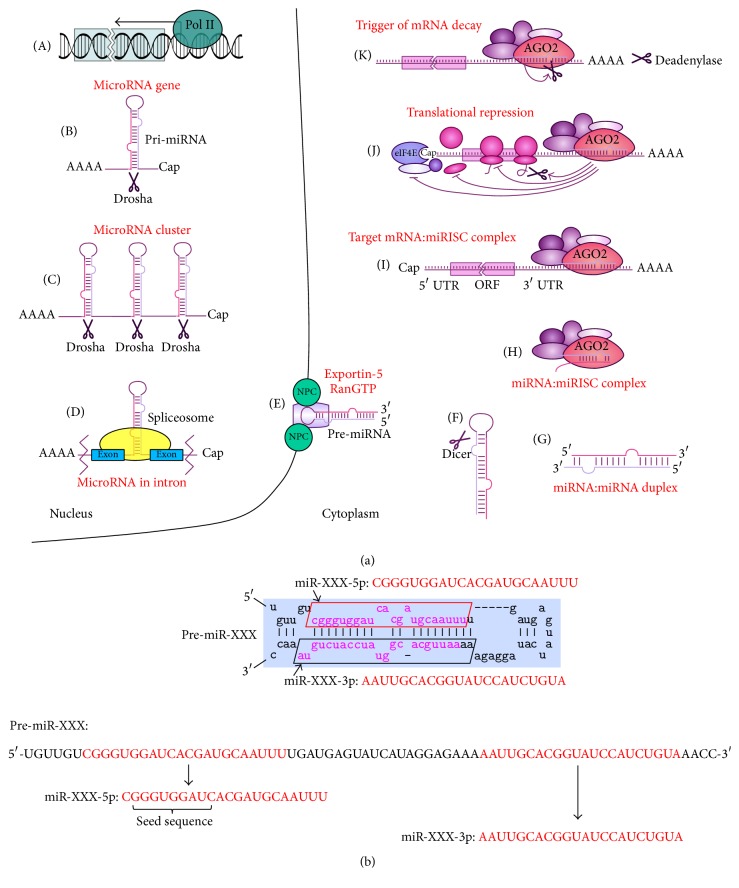
Biogenesis and mechanism of action of miRNAs. (a) miRNAs are transcribed mainly by polymerase II (A) from a gene encoding a single miRNA (B), or from a polycistronic gene (C), or from a gene in an intronic region (D). Resulting pri-miRNAs are processed by type III RNase Drosha. The newly formed stem-loop structure, pre-miRNA, is recognized by the XPO5, RanGTP complex, and is transported to the cytoplasm by exportin-5 (E). Dicer cleaves the loop (F), leaving a double-stranded fragment, the miRNA-3p:miRNA-5p duplex (G). The duplex is then unwound and loaded into the miRISC complex (H) where it recognizes and anneals to the UTR of mRNA target (I). The messenger RNA:miRISC complex mediates translational repression (J) or mRNA decay (K). (b) Processing of a pre-miRNA gives rise to two mature miRNAs named miR-XXX-3p and miR-XXX-5p where miR-XXX-3p miRNA originates from 3′-end and miR-XXX-5p miRNA originates from 5′-end of the pre-miRNA.

MicroRNAs encoding genes are located both in intronic and in exonic regions of the genome. The processing of a pre-miRNA into mature miRNAs results in two 19–23 nt long miRNAs named miR-XXX-5p and miR-XXX-3p; the mature miR-XXX-5p miRNA originates from 5′-end and miR-XXX-3p originates from 3′-end of the pre-miRNA (Figure 1). MicroRNAs may be transcribed from individual genes or as clusters [9]. Approximately 30% of miRNAs are transcribed as polycistronic clusters [10–12]. Transcription may be regulated either by its own promoter or by a host gene promoter [13]. A cluster of miRNAs is defined as several miRNA genes located adjacent to each other on the chromosome, which are transcribed as one long pri-miRNA transcript and subsequently processed into the individual pre-miRNAs [14]. The genomic organization of miRNAs in a cluster may function to protect it from degradation as the secondary structure of a longer pri-miRNA is complex with numerous hairpins that stabilize the RNA [15]. This arrangement may have particular significance as regards regulation of gene expression. Clustered miRNAs with similar sequences may regulate a set of mRNA targets and therefore function as powerful regulators of specific cellular activities. MicroRNA clusters are often transcribed by a common promoter [16, 17] and range from <100 base pairs (bp) to 50 kilobases (kb) [14, 15]. MicroRNAs within a cluster are often, but not always, paralogous with high sequence homology. This suggests that microRNAs are the result of genomic duplications [18, 19]. High sequence homology between the miRNAs in a cluster classifies them as a family and permits both common and unique mRNA targets. These mRNA targets are often present within the same pathway, allowing these miRNAs to have regulatory influence over several components of a cellular process. Consistent with this role for miRNA clusters, several clusters have been found to be important for normal development and disease pathology [20–24].

There is a close link between deregulation of normal developmental processes and tumorigenesis, and increasing evidence indicates that changes in expression of miRNAs is entailed in the pathogenesis of human cancers [25–29]. Investigating how miRNA families are expressed in clusters and how they control cell-signaling pathways is likely to increase the knowledge of cancer progression. Studies in mouse and human cells have identified the miR-17-92 cluster (also called oncomiR-1) as a potential oncogene [30]. The functional analysis of miR-17-92 is difficult because of existence of two paralogues: miR-106a-363 and miR-106b-25. The present review discusses the characteristics and functions of the three paralogous clusters miR-17-92, miR-106a-363, and miR-106b-25. These clusters are highly conserved across species [31]. It is believed that they have arisen from genetic duplications and have been found to be prooncogenic in a wide range of malignancies [32–37]. Hence, manipulation of these clusters or their component miRNAs is most likely critical for further understanding of tumorigenesis.

## 2. Biological and Oncogenic Role of the miR-17-92 Cluster

**Figure 2:**
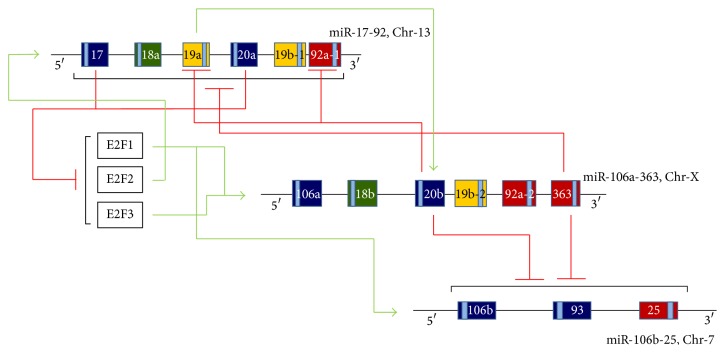
Schematic illustration of possible regulatory interactions between miRNAs encoded by the three paralogue clusters and with the E2F transcription factor family. Stimulatory effects are shown using green lines; inhibitory effects are shown using red lines.

Among the three polycistronic, paralogue clusters (miR-17-92, miR-106a-363, and miR-106b-25), the miR-17-92 cluster is so far the most studied. The miR-17-92 cluster is located on chromosome 13 in open reading frame 25 (C13orf25) in the human genome and on chromosome 14 in the mouse genome [32, 34, 38]. The primary transcript encodes six mature miRNAs: miR-17, miR-18a, miR-19a, miR-19b-1, miR-20a, and miR-92a-1 (Figure 2, Table 1). These are encoded within 800-base-pair region in the human genome. The six miRNAs can be grouped into four miRNA families based on their seed-sequence: the miR-17 family (miR-17 and miR-20a), the miR-18 family (miR-18a), the miR-19 family (miR-19a and miR-19b-1), and miR-92 family (miR-92a-1) [31, 34, 39].

The oncogenic potential of miR-17-92 cluster was first described by He and colleagues [38] and has been observed both in human tumors and in animal models. This cluster is deleted in ovarian, breast, and skin cancers [40] but amplified in human lymphomas [34]. It has also been described as a common retroviral insertion site [34]. Members of the miR-17-92 cluster are expressed in a variety of tissues, although the effect of these miRNAs depends on the cellular context. For example, in different murine embryonic tissues individual miRNAs were highly expressed at an early stage of the development, whereas they abated at later stages [41]. Several studies where the cluster, or individual members of the cluster, has either been overexpressed or deleted have shown essential functions of this cluster both in development and in disease. Ventura and associates reported that mice deficient for miR-17-92 died shortly after birth with lung hypoplasia and a ventricular septal defect [42]. In the same study the authors demonstrated that the miR-17-92 cluster is also essential for B cell development. Deletion of the miR-17-92 resulted in enhanced levels of the proapoptotic protein BIM and inhibition of B cell development at the pro-B to pre-B transition. Report from another group demonstrated the involvement of this cluster in development of cerebellar and medullar blastoma [43].

Abnormal expression of members of the miR-17-92 cluster in cancer indicates that these miRNAs are involved in carcinogenesis. They may function either as oncogenes (oncoMIRs) or as tumor suppressors, dependent on their target genes [38, 39, 42, 44–47]. While overexpression of the oncogenic miR-17-92 cluster has been identified in many cancers, researchers also observed that this cluster could act as a tumor suppressor in certain cancer types. This cluster was found to be deleted in 17% of ovarian cancers, 20% of melanomas, and 22% of breast cancers; for example, miR-17-5p has tumor suppressor role in breast cancer by repressing the expression of AIBI and Cyclin D1 [48–50].

MicroRNAs encoded by the cluster have been found to target genes with important functions in cell cycle progression and apoptosis and in angiogenesis [44, 47, 51–54]. The E2F family of transcription factors, which when expressed at high levels may induce apoptosis, is one such target [47]. The miR-17-92 cluster miRNAs, therefore, may suppress apoptosis by downregulating E2F1, E2F2, and E2F3 (Figure 2). The proapoptotic gene BIM is also a direct target of miR-92a [55, 56]. Another target that has been validated for miR-17-5p is cyclin-dependent kinase inhibitor p21, which is a negative regulator of the G1/S checkpoint [57]. All members of miR-17-92 cluster, except miR-18, are also known to downregulate expression of the tumor suppressor PTEN [52]. Furthermore, the antiangiogenic proteins TSP11 and CTGF are both negatively regulated by miR-18 and miR-19 [58]. High levels of members of the miR-17-92 cluster have been reported to increase the number of leukemia stem cells, block differentiation, and enhance proliferation, while low levels of the miR-17-92 cluster increase differentiation and diminish self-renewal of stem cells [44, 59–61].

Members of the miR-17-92 cluster have been shown to be involved in regulation of transforming growth factor-*β* (TGF-*β*)/SMAD signaling, a pathway with critical role in cell growth, differentiation, and development in many cellular systems [62–65]. miR-17 and miR-20a have been identified to target TGFBRII [66, 67]. It is suggested that SMAD2/4 is regulated by miR-18 in neuroblastoma cells [66] and that SMAD4 is targeted by miR-19a/b in thyroid follicular cells [68]. In addition, it has been demonstrated that miR-18 and miR-19 repress the antiangiogenic factors TSP-1 and CTGF [51]. miR-17, miR-20a, and miR-92 also illustrated the importance of collaboration in the regulation of Isl1 and Tbx1 during cardiac development [69].

## 3. The Role of miR-106a-363 and miR-106b-25 Clusters in Development and Disease

The miR-106a-363 cluster is located on chromosome X in mice and humans. This cluster encodes six miRNAs: miR-106a, miR-18b, miR-19b-2, miR-20b, miR-92a-2, and miR-363 [42]. miR-20b is reported to be up- or downregulated in different cancers [70–73]. Decreased expression of miR-363-5p was detected in head and neck carcinomas and breast cancer cell lines [74, 75]. Tumors with low levels of expression of miR-363-3p or miR-363-5p, as well as high levels of expression of B7-H3 or E2F4, were associated with lower probability of survival [74, 76, 77]. Moreover, the miR-363-5p regulates angiogenic properties of endothelial cells as well as their communication with hematopoietic precursor cells. miR-363-5p is shown to regulate the expression of angiocrine factors tissue inhibitor of metalloproteinases-1 (Timp-1) and thrombospondin 3 (THBS3). Blocking the expression of miR-363-5p was shown to affect endothelial cell response to angiogenic factors stimulation [78].

The miRNA derived from the complimentary strand of miR-363-5p pre-miR, miR-363-3p, has been shown, depending on the type of cell involved, to have dual functions either as tumor suppressor or as oncogenic miRNA. A high level of miR-363-3p suppresses proliferation of human hepatocellular carcinoma cells by targeting S1PR1 or USP28 [79, 80]. This high level has also been associated with diminished metastasis of human neuroblastoma cell lines BE(2)-C and SK-N-SH by regulating expression of ADAM15 and MYO1B in these cell lines [81]. This was also observed in head and neck squamous cell carcinomas; miR-363-3p inhibits expression of the transmembrane glycoprotein podoplanin [75]. The miR-363-3p has also been proposed to regulate the transition from mitotic clonal expansion to terminal differentiation during adipogenesis in adipose tissue-derived stromal cells (ADSCs) by targeting E2F3 [82].

Additionally, miR-363-3p has been shown to exhibit an opposite effect in gastric cell lines; knock-down of expression of miR-363-3p was found to suppress carcinogenesis in certain gastric cancer cells (SC-M1-, KATO III-, and SNU-16) by upregulation of MBP-1 [83]. An intriguing study by Wagh et al. [84] demonstrated the critical role of miR-363 in posttranscriptional regulation of cardio myocyte differentiation by targeting the cardiac transcription factor HAND1, necessary for the development of left ventricle of the heart. Here, overexpression of miR-363-3p resulted in downregulation of amount of both HAND1 mRNA and protein.

Our group has shown that both miR-20b and miR-363 from this 106a-363 cluster are barely detectable in human oral carcinoma cell line E10 [85]. Overexpressing E10 cell line with miR-20b mimic led to reduced proliferation. Furthermore, transfection with miR-363-5p mimic led to diminished expression of miRNA members from miR-17-92 and miR-106b-25 cluster, which also resulted in reduced proliferation of E10 cells [85].

The miR-363-5p is also expressed in aged oral keratinocytes [85]. Other studies reported that the level of miR-363-5p was either increased or decreased in aged mice [86]. Our results, together with published data, indicate that expression of members of the miR-106a-363 cluster is cell specific. Also, a miRNA derived from either the 3′- or 5′-strand may be active as shown for miR-363, although only one of the two strands is expressed at any given time [85].

The miR-106b-25 cluster is located in the 13th intron of DNA replication gene Mcm7, which resides on chromosome 7 in humans and on chromosome 5 in mice. The cluster encodes three miRNAs: miR-106b, miR-93, and miR-25 [35, 39]. Both the evolutionary sequence analysis and the seed-sequence-based grouping partition these miRNAs into four families: the miR-106 family (miR-17, miR-20a/b, miR-106a/b, and miR-93), the miR-18 family (miR-18a/b), the miR-19 family (miR-19a/b-1/2), and the miR-92 family (miR-25, miR-92a-1/2, and miR-363). The miRNAs within each family exhibit substantial sequence homology outside of their seed-sequences [35].

Members of miR-106b-25 cluster are overexpressed in several cancers including gastric cancer [87], hepatocellular carcinoma [88], esophageal adenocarcinoma [89], neuroblastoma [90], and prostate cancer [91]. Like the miR-17-92 paralogue, miR-106b-25 is likely prooncogenic. Overexpression of members of the miR-106b-25 cluster has been shown to increase cell proliferation and anchorage-independent growth [88]. In prostate cancer, miRNA encoded by this cluster has been shown to target the tumor suppressor PTEN [71, 91] and to cooperate with its host gene (MCM7) to increase tumor growth in mice [91]. Among other cellular targets described for miR-106b-25 encoded miRNAs are several tumor suppressors including BIM, p21, and E2F1 [35, 89, 91, 92]. The association of this miRNA cluster with these targets has been clearly demonstrated in prostate cancer, esophageal adenocarcinoma, and gastric cancer [35, 87, 89, 91]. E2F1 is a strong stimulator of transcription of Mcm7 gene, which also encodes the miR-106b-25 cluster. Translation of E2F1 is inhibited by miR-106b and miR-93 (derived from this cluster). This creates a negative feedback loop between E2F1 activity, miR-106b/miR-93, and transcription of the Mcm7 gene.

The role of miRNAs encoded by the miR-106b-25 cluster has also been linked to growth and maintenance of stem/progenitor cells. High expression of these miRNA was found in bronchioalveolar stem cells from mouse lung [93], neuronal stem/progenitor cells [94], and nephron progenitors [95]. These miRNAs were shown to promote proliferation and maintenance of the bronchioalveolar stem cell pool and to increase survival of nephron progenitor cells through repression of BIM. Additionally, these miRNAs can promote reprograming of mouse embryonic fibroblasts to induced pluripotent stem cells [96].

Since miR-17-92 and miR-106-25 clusters show high degree of sequence similarity, it is not surprising that these two clusters share the ability to regulate the same pathways or the same genes. Indeed, miR-106b-25 cluster is shown to regulate the TGF-*β* pathway by targeting Six1 and Smad7, which are required to activate TGF-*β* pathway from suppressive to supportive tumor growth in human breast cancer [97–99].

## 4. Conclusions and Future Directions

To functionally understand the biogenesis of mature miR-17-92 miRNAs, it is important to integrate transcriptional and posttranscriptional regulatory mechanisms. Furthermore, after primary transcription of miR-17-92, its posttranscriptional control is accountable for fine-tuning the generation of miR-17-92 miRNAs. The molecular tertiary structure of the miR-17-92 primary transcript emerges as a significant modulator of miRNA processing machinery but does not fully state the distinct patterns in expression of miR-17-92 components as observed experimentally [100–102]. Therefore, it is possible that the RNA-binding proteins play a role in selectively targeting and regulating miRNAs of the cluster during processing.

Since the miRNAs encoded by the three clusters are highly similar in sequence, they may also exhibit overlapping functions. To address this issue, Ventura et al. [42] deleted the clusters in mice, while preserving the expression of the Mcm7 gene. Individual deletion of either the miR-106a-363 or the miR-106b-25 cluster caused no obvious abnormalities. The mice remained viable and fertile. In contrast, mice lacking miR-17-92 expression died soon after birth, due to lung hypoplasia and cardiac ventricular septal defects. Double knockout of miR-17-92 and miR-106b-25, or the triple knockout, resulted in more severe defects, with death at midgestation [42]. There are many possible explanations for these observations. For example, expression of these three clusters could be spatially and temporally segregated. This is likely the case for miR-106a-363 that appears to be expressed at much lower levels compared to the other two clusters [85]. However, miR-106b-25 and miR-17-92 are very similar in terms of both expression levels and tissue distribution. One possible relevant difference between these two clusters is that miR-17-92, but not miR-106b-25, expresses members of the miR-19 and miR-18 families. It is tempting to speculate that loss of miR-19a, miR-19b, and miR-18 is significantly responsible for the phenotype caused by deletion of miR-17-92.

Understanding the biological role of miR-17-92 cluster is essential for translating knowledge from bench to bedside. In vitro studies revealed antitumorigenic effects of targeting miR-17-92 in cancer cell lines. Furthermore, in vivo animal models have shed light on the potentiality of targeting miR-17-92 components therapeutically. The use of intravenous delivery of anti-miR-17-92 for the treatment of allograft medulloblastoma tumor in immune-compromised mice resulted in blockage of tumor growth [103], indicating miR-17-92 as a potential therapeutic target. Some issues, however, regarding selective anti-miR delivery to cancer cells and its side effects in animal models still need to be addressed by further studies in order to permit the safe application of anti-miR-17-92 as a therapeutic adjuvant for treatment of cancer.

Although we have significantly increased our knowledge about the role of miR-17-92 cluster in development and cancer during the past years, we still have to reveal the extensive regulatory mechanisms this cluster and its two paralogues have in mammals. Thorough investigations on the individual functions of the miR-17-92 members towards different biological contributions will be fundamental to understand the degree of functional overlap and interworking between the members of these miRNA clusters. The generation of different mouse models combined with new approaches to study miRNA-mRNA interactions is warranted to fully answer these essential questions. In conclusion, there are both important and overlapping functions for these three paralogous miRNA clusters. More functional analysis will provide new insights into the regulation of crucial developmental programs by miRNAs and indicate a supplemental level of regulation by this class of molecules in the form of functional overlap.

## Figures and Tables

**Table 1:** The role of the three paralogous miRNA clusters in development and disease.

MicroRNA cluster	Chromosome	MicroRNAs	Biological functions	Oncogenic role
miR-17-92	13	miR-17, miR-18a, miR-19a, miR-19b-1, miR-20a, and miR-92a-1	Angiogenesis [51, 104] Apoptosis [47, 105, 106] B cell development [38, 42, 107] Cell cycle progression [47, 105, 106] Development of lung, heart, kidney, cerebral hemisphere, small intestine, submandibular salivary gland, and tooth germ [41, 42, 108] Enhanced proliferation [42, 44, 85] Inhibited differentiation [59]	*Expression in human*: Colon cancers ↑ [56] Lung cancers ↑ [44, 59] Lymphomas ↑ [47] Solid tumors ↑ [40] Thyroid cancers ↑ [109, 110] Tumor angiogenesis [51, 104]

miR-106a-363	X	miR-106a, miR-18b, miR-19b-2, miR-20b, miR-92a-2, and miR-363	Aging [111] Angiogenesis [78] Apoptosis [112] Cellular growth [79, 85] Development of lung and heart [84]	*Expression in human*: Ewing sarcoma tumors ↓ [113] Head and neck cancers ↓ [75] Oral cancers ↓ [85] T cell leukemia ↑ [32]

miR-106b-25	7	miR-106b, miR-93, and miR-25	Apoptosis [87] Cell cycle progression [32] Proliferation and differentiation [94]	*Expression in human*: Esophageal cancers ↑ [89] Gastric cancers ↑ [87] Hepatocellular tumors ↑ [88] Prostate cancers ↑ [32]
